# Artificial Intelligence in Oral and Maxillofacial Surgery—A Narrative Review

**DOI:** 10.3390/jcm15176728

**Published:** 2026-08-30

**Authors:** Dominika Zawadka-Modras, Jacek Rożko, Aldona Chloupek, Dariusz Jurkiewicz

**Affiliations:** 1 Clinical Department of Cranio-Maxillofacial Surgery, Clinic of Otolaryngology and Laryngological Oncology, Military Institute of Medicine—National Research Institute, 04-141 Warsaw, Poland; jrozko@wim.mil.pl (J.R.); achloupek@wim.mil.pl (A.C.); 2 Clinical Department of Otolaryngology, Military Institute of Medicine—National Research Institute, 04-141 Warsaw, Poland; djurkiewicz@wim.mil.pl

**Keywords:** artificial intelligence, large language models, convolutional neural networks, dentistry, maxillofacial surgery, oral surgery, AI

## Abstract

**Background/Objectives**: Artificial intelligence (AI) is rapidly transitioning from a theoretical concept to an active clinical tool in dentistry and medicine. These are fields in which the current market-driven shift towards automation is observed. The aim of this study was to describe the possible applications of artificial intelligence particularly in oral surgery and maxillofacial surgery, fields that are deeply rooted in manual work with patients. **Methods**: A review of current literature was conducted using PubMed/MEDLINE, Scopus, and Web of Science databases. Keywords like: “oral surgery”, “maxillofacial surgery”, “implantology”, “dentistry”, “artificial intelligence”, and “orthognathic surgery” and their combinations were applied. Non-English articles were excluded. **Results**: Based on the literature, current applications of artificial intelligence in oral surgery and maxillofacial surgery are presented. Results indicate high efficacy of convolutional neural networks (CNNs) in radiographic triage and large language models (LLMs) in postoperative patient communication. **Conclusions**: Artificial intelligence is a useful tool that can significantly improve the work of physicians. However, it should not be considered a “replacement” for physicians, especially in fields requiring manual work. It can provide important guidance for patients and be a form of support for physicians.

## 1. Introduction

Artificial intelligence (AI) has rapidly transitioned from a theoretical concept to an integral component of modern healthcare. Dentistry is no exception; the clinical implementation of AI is accelerating significantly. The integration of AI is frequently perceived as a hallmark of modern clinical practice. This is primarily seen in dental radiology [1], but also in restorative dentistry, endodontics, periodontics, prosthodontics, orthodontics, and finally, dental surgery, maxillofacial surgery, and implantology. The use of AI begins at the stage of tooth numbering on X-rays [2], through caries diagnostics [3] in both permanent and deciduous teeth [4], the planning of prosthetic restorations [5], and formulating orthodontic treatment plans. It also encompasses the entire branch of surgery. It can be utilized in basic procedures like tooth extraction, in implantology, for diagnosing periapical and periodontal lesions [6], in oncology and orthognathic surgery, as well as for patient education. As AlFarabi Ali [7] demonstrated, the effectiveness of AI in diagnosing patients based on clinical scenarios reaches 74%, and there are no significant differences in the accuracy of making correct differential diagnoses between artificial intelligence models and dentists.

The prototypes used in various medical applications are complex neural networks capable of supervised and unsupervised learning [8]. This means that a network can be trained for specific applications that were previously chosen, but it can also search for new patterns based on provided data. To fully appreciate their clinical utility, it is essential to distinguish between the main subtypes of artificial intelligence employed. In oral and maxillofacial surgery, convolutional neural networks (CNNs) are the foundational architecture for processing visual data. They act as the “eyes” of the system and are utilized primarily for radiological imaging analysis, automated anatomical landmark detection, and tumor or lesion segmentation. Conversely, large language models (LLMs), built on natural language processing, are increasingly employed for text-based tasks. They serve to evaluate complex medical histories, streamline clinical documentation, and support patient education and postoperative care through interactive chatbots. Finally—and looking toward the future—multimodal AI models represent the pinnacle of achievement in this field. These systems could simultaneously analyze multiple data types—such as combining a 3D cone beam computed tomography (CBCT) scan (visual) with a patient’s HbA1c levels and medical history (text/tabular)—to generate comprehensive, personalized risk assessments. Integrating medical imaging, genomic data, and patient notes enhances diagnostic accuracy and precision medicine. Interestingly, although dentists recognize the positive aspects of using AI in modern diagnostics and treatment [9,10], they usually do not use this technology very regularly and the use of AI itself is inversely correlated with the dentist’s age [11,12]. The above factors suggest conducting research in this field and categorizing the use of artificial intelligence in selected areas of dental and maxillofacial surgery.

Filling this interdisciplinary gap, the narrative review was chosen as a form of synthesis of the application of AI in oral and maxillofacial surgery. Specific demands of this field of medicine allowed us to summarize the current state of knowledge, citing the data but at once noting the limitations of the evidence base.

## 2. Materials and Methods/Literature Search Strategy

### 2.1. Search Strategy and Databases

A comprehensive literature search was conducted to inform this narrative review, with the primary aim of identifying publications relevant to the application of artificial intelligence in oral and maxillofacial surgery (OMFS). To ensure a broad overview of the current literature, electronic searches were conducted across three major databases: PubMed/MEDLINE, Scopus, and Web of Science. The search strategy utilized combinations of Boolean operators (AND/OR) with the following keywords and search terms: (“artificial intelligence” OR “large language model” OR “convolutional neural network”) AND (“oral surgery” OR “maxillofacial surgery” OR “implantology” OR “dentistry” OR “oncology maxillofacial surgery” OR “orthognathic surgery” OR “dental foci”). The final search update was performed in May 2026.

### 2.2. Inclusion of Gray Literature

To improve the clinical relevance and transparency of this review, targeted gray literature was also included. Specifically, official public health reports published by the UK National Health Service (NHS) regarding histopathology turnaround times were manually retrieved, as well as Recommendations for a Predetermined Change Control Plan for Artificial Intelligence-Enabled Device Software Functions published by the Food and Drug Administration (FDA).

The inclusion of this gray literature was deemed highly relevant and essential, as real-world diagnostic delays provide critical context for why AI-driven histopathological analysis is currently needed in maxillofacial oncology, while the FDA recommendations represent an essential step toward the clinical implementation of artificial intelligence.

### 2.3. Eligibility Criteria and Study Selection

The selection of studies was guided by predefined inclusion and exclusion criteria. Studies were included if they: (1) addressed AI applications directly within oral or maxillofacial surgery; (2) provided clear references to AI improving the surgical workflow (e.g., comparing workflows with and without AI support); or (3) contributed relevant conceptual insights from general medicine or bordering disciplines, such as periodontology, that are integral to comprehensive maxillofacial care.

The search focused primarily on articles published between January 2018 and May 2026, reflecting the rapid recent evolution of AI in medicine. Older, landmark papers (published prior to 2018) were retained only when considered essential for historical context. Articles published in languages other than English, studies lacking clinical relevance to OMFS, and papers focused exclusively on unrelated dental sub-disciplines were excluded.

### 2.4. Screening Process and Record Yield

The initial database search yielded approximately 3500 records. After the removal of duplicates, the titles and abstracts of approximately 900 records were screened for relevance. Subsequently, the full texts of 220 promising articles were retrieved and thoroughly assessed for eligibility. Reference lists of key publications and recent reviews were also manually screened to identify additional relevant studies. Following this selection process, a final total of 74 references were included in this review. Priority was given to clinical studies, prospective cohorts, case series, technical descriptions, and biologically oriented conceptual papers.

### 2.5. Methodological Considerations

Due to the narrative and non-systematic design of this review, a wide variety of studies was included and an overall summary was provided. At the same time, formal systematic-review methodology, risk-of-bias assessment, certainty-of-evidence grading, and meta-analytic synthesis were not performed. Consequently, the conclusions should be interpreted as a structured but still subjective overview across various fields of oral and maxillofacial surgery rather than as evidence-based clinical recommendations. This methodological choice allowed the integration of different ways of applying AI and emerging clinical reports from all fields connected with oral and maxillofacial surgery. Taking into consideration that artificial intelligence is a rapidly evolving field, the narrative character of this manuscript allowed knowledge to be kept up to date. However, it is worth emphasizing that this is an intentionally narrative and therefore non-exhaustive review. This review does not prioritize the technical aspects of the matter and carries an inherent risk of selection bias associated with this study design, but provides an overview of the topic and encourages consideration of implementing artificial intelligence in the medical practice, drawing attention to its possibilities for application.

## 3. Review of AI Applications: A Clinical Workflow Perspective

To systematically evaluate the integration of artificial intelligence in oral and maxillofacial surgery, it is highly practical to categorize its applications according to the clinical workflow. Regardless of the specific surgical sub-discipline—whether routine dentoalveolar extractions, complex implantology, or extensive maxillofacial oncology—the patient journey consistently follows three distinct phases: preoperative preparation, intraoperative execution, and postoperative management.

Different AI architectures are uniquely suited to specific tasks within these phases. CNNs predominantly occupy the preoperative and intraoperative stages with augmented reality (AR) due to their superior image recognition and segmentation capabilities (e.g., anatomical auto-segmentation, cephalometrics, and real-time margin visualization). Meanwhile, LLMs and predictive multimodal AI systems are proving increasingly invaluable for patient triage, post-surgical communication, and long-term risk assessment. The following sections explore these targeted applications in detail across various domains of oral and maxillofacial surgery.

In related fields such as otolaryngology, hybrid CNN–LLM frameworks have shown strong diagnostic potential, with recent retrospective single-center evaluations reporting up to 97.6% diagnostic accuracy in otoscopic exams [13]. However, because performance metrics from individual studies are heavily influenced by curated cohorts and data acquisition protocols, these findings must be interpreted with caution until validated in real-world multi-center settings. Nevertheless, these multimodal architectures represent a promising future direction for complex maxillofacial triage.

### 3.1. Oral Surgery

Based on the literature review, the applications of artificial intelligence in dental surgery were categorized as follows.

#### 3.1.1. Tooth Extractions

The fundamental procedure in dental surgery—tooth extraction—can be supported by artificial intelligence at every stage of the procedure, i.e.,

- preoperatively—for procedure planning, identifying possible complications, and risk estimation;
- intraoperatively—at the execution stage, improving surgical precision;
- postoperatively—maintaining patient contact and providing postoperative instructions.

##### Preoperative Stage

It seems that currently, the most important capability that may be utilized is radiological analysis. Conventional radiological interpretation is undoubtedly a necessary and indispensable element of clinical work; however, it is subject to perceptual errors and the individual clinical experience of the evaluator, whereas AI models are trained on an infinitely larger number of images [14], which would take a human years to review. Naturally, the clinical experience of a veteran radiologist still significantly surpasses the skills of artificial intelligence. Nevertheless, it is worth considering AI X-ray analysis as a form of preliminary radiological assessment, a tool for young doctors, and an educational element for patients.

A key extraction procedure is the removal of an impacted third molar. The indications for removing such a tooth, the probability of its eruption in the oral cavity, and the possible complications after third molar extraction—namely inferior alveolar nerve injury [15]—remain subjects of discussion. Using artificial intelligence, the mandibular canal, the root morphology of the third molar, and the surrounding bone—based on a CBCT scan—may be segmented. By creating such models, the quantitative data regarding the tooth-to-nerve-canal relationship may be obtained, which makes the classic, subjective assessment of the risk of inferior alveolar nerve injury more objective [16] and based on quantitative data. For the patient, numerical values are more understandable and precise, ensuring that the decision to undergo the procedure and accept the surgical risk is backed by “hard” data.

Before the patient decides on the procedure, the data evaluating the “difficulty index” of the surgery can also be provided. Root morphology, possible dilaceration, angulation relative to the adjacent tooth, and the density of the surrounding bone can then be taken into account. Such an “index” can also assist dentists of other specialties in qualifying third molars for potential extraction before referring them to a specialist. For specialists, it can help categorize specific teeth for multiple extractions, coronectomies, or single extractions—in short, a standardized extraction “difficulty index” [17,18] can help appropriately plan surgical time (often scheduled by receptionists), accurately price the procedure at the booking stage, and allow the patient to properly plan their recovery time regarding work and family life. Serap Akdogan [19] developed an AI-supported clinical tool for assessing the difficulty of mandibular third molar extractions based on panoramic radiographs.

Thus, retrospectively assessing the developmental stages, the answer to the patient’s question: “Is there a chance this tooth will erupt?” [20] may be given. Of course, there are several described indices to estimate the probability of such an eruption; however, accurately determining and calculating this takes time. AI saves the doctor time while providing the patient with a more systematized, quantitative answer. Consequently, the decision regarding potential extractions becomes more predictable; an orthodontist, in consultation with a surgeon, can also plan such extractions earlier, avoiding patient dissatisfaction when orthodontically indicated extractions must be performed immediately after brace removal.

Furthermore, the “decision stage” itself can be supported by chatbots based on large language models (LLMs). Şişman AÇ [21] examined the factual accuracy of answers provided by ChatGPT 3.5 (by OpenAI, San Francisco, CA, USA) and Claude (by Anthropic, San Francisco, CA, USA) to questions concerning maxillofacial surgery topics. The quality, accuracy, and completeness of the responses were evaluated by two specialists. LLMs achieved over 90% accuracy and over 85% completeness of responses, respectively. These tools can provide quick solutions amid the growing demand for specialists, reduce the employment costs of highly qualified staff, and save their time.

##### Intraoperative Stage

Although still in the early stages of implementation compared to preoperative tools, intraoperative AI is set to revolutionize the technical execution of extractions. Similar to navigated implantology—where this solution is already more widely used—the surgical guides for complex extractions can be utilized. However, artificial intelligence offers something more: dynamic navigation [22]. Proximity of the surgical drill to critical anatomical structures, such as the inferior alveolar nerve canal, maxillary sinus, lingual plate, or adjacent roots, can trigger real-time audiovisual alerts. An ideal “safety zone” [23] for a given patient may be predefined, thereby increasing the predictability and safety of the procedure.

Moreover, an augmented reality (AR) overlay provides all of the above not on a separate screen or as sound, but directly “on the patient.” This means the X-ray view is superimposed directly onto the patient’s surgical site. Thanks to this, the performed osteotomy can be precise, careful, and in line with the “less is more” philosophy [24]. It is worth to add that this application of AI including AR is rather a possibility of the future than the common practice.

The ultimate stage of advancement seems to be robotic surgery, which is already widespread in gynecology, urology, general surgery, ENT, and neighboring maxillofacial surgery. While this seems like a distant prospect, the surgeon would eventually only map the extent of the osteotomy, which would be executed by a robot, eliminating issues like hand tremors. The key limitation today is economics, which currently prevents robotic surgery from finding a practical application in tooth extractions. Therefore, this remains rather a future perspective than a current trajectory.

##### Postoperative Stage

A surgeon’s responsibility does not end when the procedure is finished. The postoperative care phase is just as important as the execution or preparation of the surgery. Artificial intelligence can also help the doctor qualitatively assess healing, i.e., compare pre- and postoperative X-rays, evaluating the degree of socket fill, the speed of its filling over time, and the state of bone tissue healing after the removal of extensive cysts when it is visually difficult to superimpose several images. It can also alert the doctor if healing is projected to fail.

Finally, there is an issue that seems downplayed but immensely impacts the patient’s peace of mind and sense of security: postoperative contact. Some doubts regarding healing only arise in the days following the surgery, and at that time, help and reassurance from the doctor are invaluable to the patient. Personalized, 24/7 chatbots can streamline communication, triage patient needs, and inform the doctor about specific cases requiring immediate attention [25].

##### Level of Evidence and Clinical Maturity

While the intraoperative dynamic navigation and augmented reality guidance rely on Level 1 case reports and feasibility trials [16] and preoperative radiographic risk assessment models rest on Level 2 retrospective data [17], the field is supported by comparative evaluations of LLM decision assistance [21,25]. Specifically, retrospective studies confirm that deep learning models significantly enhance third-molar risk prediction and extraction difficulty categorization, though larger Level 3 prospective clinical trials are needed to drive tooth extraction AI into routine clinical implementation (Level 4).

#### 3.1.2. Implantology

Implantology has long been at the forefront of digital innovation, starting with the transition from 2D to 3D imaging, and then the use of intraoral scanners instead of traditional impressions. The next step appears to be the integration of AI into implant treatment. The goal is to support the doctor’s planning and provide tools that make this process even more predictable, calculated, safe for the patient, and time-efficient.

Implant treatment and AI integration can also be divided into three phases:

- preoperative stage—diagnostics and planning of the surgical procedure and prosthetic restoration;
- intraoperative stage—execution of the surgical procedure;
- postoperative stage—postoperative monitoring of the patient and the implant-prosthetic restoration.

##### Preoperative Stage

Automatic identification of anatomical landmarks performed by AI immediately upon opening a CBCT scan significantly shortens the preparatory phase. Spontaneous identification of structures such as the inferior alveolar nerve canal, the floor of the maxillary sinus, nasal cavity walls, mental foramina, or the mandibular incisive canal [26] is crucial for safely planning an implant procedure. This significantly reduces preparation time, eliminating a tedious, mathematical task and, more importantly, acting as a safety net to minimize iatrogenic nerve damage or maxillary sinus membrane perforation.

Based on CBCT, a detailed analysis of the planned implant site, i.e., a three-dimensional bone map with calculated bone density based on the Lekholm and Zarb model can be received. This can influence implant selection, such as choosing a more aggressive thread or a specific drilling technique for soft bone.

Finally, artificial intelligence can integrate data available on CBCT with general medical history data, factoring in elements like HbA1c levels, smoking history, and periodontitis. The model can generate a patient-specific risk index for osseointegration failure or peri-implantitis. Simply familiarizing the patient with these expected difficulties preoperatively significantly increases surgical comfort for the surgeon and later helps manage potential future complications [27]. This ability to predict complications marks the beginning of the “personalized medicine” chapter. At-risk patients can be scheduled for more frequent follow-up visits or subjected to a more conservative prosthetic loading protocol.

Regarding the planning of the prosthetic restoration itself, the concept of “crown-down” (restoration-driven) implant planning currently dominates implantology. Artificial intelligence is an excellent tool for optimizing this technology. The classic combination of CBCT with STL (Standard Triangulation Language) files obtained from a scan allows for generating the most optimal virtual surgical-prosthetic configuration. It considers surgical factors such as the distance from key anatomical structures and position relative to the bone crest, as well as prosthetic factors like ensuring a proper emergence profile, esthetic screw access, and correct loading while minimizing off-axis forces. The doctor immediately receives several individualized plans, and the planning time itself is significantly reduced—from about 30 min per case to 10 min [7].

Another advantage, which has actually been utilized in some form for years, is helping the doctor identify the brand of an implant [28,29] they did not place. Patients today are very mobile; most move several times in their lives, which in a dental context means that the surgery and prosthetic restoration are not always performed by the same person, or at least not in the same clinic. Studies show that the diagnostic accuracy of AI implant brand recognition ranges between 67% and 99% [30]. The multitude of implant companies on the market ensures that such an application of artificial intelligence will certainly find many users.

##### Intraoperative Stage

At this stage, a surgical guide prepared by AI after approving the selected implant-prosthetic plan or performing the surgery in augmented reality can be utilized [31]. As with tooth extractions, the use of robotic surgery could contribute to executing a maximally precise osteotomy, thereby eliminating hand tremors. It is worth noting, however, that—primarily for economic reasons—this method, particularly when involving AR, remains the exception rather than the rule in implant procedures.

##### Postoperative Stage

Artificial intelligence can support treatment activities not only pre- and intraoperatively but also postoperatively. By comparing X-rays, the level of osseointegration may be assessed and the mesiodistal distance of the placed implant may be evaluated—which is crucial for its prognosis [32]—and abnormalities could also be detected earlier, such as changes in bone density around the implant. Thus, earlier intervention, like removing occlusal overload or off-axis forces, can protect the patient from peri-implantitis and, consequently, from implant loss [33].

Moreover, as mentioned earlier regarding tooth extractions, a chatbot seems to be an excellent tool, primarily because it is available 24/7. The platform can contribute to further educating patients in dental implantology and helping them understand treatment procedures. However, it is concerning that AI may exhibit bias regarding dental implant brands, which could influence patient advice [34].

##### Level of Evidence and Clinical Maturity

Implantology AI applications demonstrate advanced clinical maturity across several workflow stages. While intraoperative augmented reality guidance relies on Level 1 proof-of-concept studies [31] and CBCT landmark segmentation and prognostic modeling rest on Level 2 retrospective data [26,32,34], the field is strongly backed by Level 3 prospective comparative trials and systematic reviews [27,30,33]. Specifically, systematic reviews confirm high accuracy in implant brand identification [28,29,30] and peri-implant bone loss detection [33], while prospective comparative trials demonstrate improved patient understanding and reduced anxiety [27], driving implantology AI close to routine clinical implementation (Level 4).

#### 3.1.3. Diagnosis and Education of Patients (And Doctors)

The beginning and end of the treatment process provide an excellent foundation for introducing artificial intelligence, namely: the beginning, associated with establishing the correct diagnosis, and the end, involving postoperative patient monitoring.

##### Preoperative Diagnostic Triage

Most patients have a positive attitude toward introducing AI as a permanent element of the treatment process. Machine learning models can be used on a “triage” basis. Algorithms can automatically detect on 2D and 3D images, after segmentation, issues such as dental caries, periapical lesions, cysts, maxillofacial fractures, temporomandibular joint (TMJ) changes, or alveolar bone loss, while simultaneously guiding early periodontitis prevention. Such “screenings” somewhat standardize the diagnostic process and increase the chances that small lesions will not be overlooked. Furthermore, they streamline workflow among doctors of different medical specialties; e.g., facial bone fractures might be noticed faster by a conservative dentist, caries by a periodontist, and bone loss by an orthodontist. AI can act as a radiological “bridge” connecting doctors of all specialties.

However, perhaps the most groundbreaking moment for the doctor–patient relationship will be precise anatomical visualization followed by surgical plan simulation. Instead of pointing a pen at another gray spot on an X-ray, AI can color-code a subgingival cavity, then highlight the inferior alveolar nerve path in yellow, the maxillary sinus space in blue, and an existing cyst or tumor in red. This significantly increases patient engagement in the treatment process, and the visual simulation serves as a valuable attachment to the complete medical record [35].

AI can be used not only for educating patients but also for educating doctors. In a study [36], 31% of lecturers are already using AI tools in the teaching process, and over 64% recognize the potential for their use. A significant difference emerges between respondents in Europe and those in the USA, Asia, and Africa; currently, significantly less potential for AI use is noted in Europe.

Furthermore, in another study, Baglivo [37] demonstrated the effectiveness of AI chatbots in answering complex medical questions, with their results significantly outperforming those of medical students. Thus, the use of AI chatbots can significantly enhance medical education.

Moreover, as reported by Chow R., artificial intelligence demonstrates comparable, if not better, effectiveness in recruiting patients for cancer clinical trials than manual screening. It requires less time and human resources for patient screening [38].

##### Postoperative Patient Monitoring

Postoperative patient monitoring is one of the most important elements of the entire treatment process. Programmed chatbots can guide the patient through the postoperative period or during multi-stage treatment; for instance, during orthodontic treatment, they have a positive impact on patients maintaining proper oral hygiene [39]. Their use also positively affects patient satisfaction [40]. The main benefits expected by patients are greater diagnostic certainty, reduced time, and more personalized, evidence-based treatment [41].

##### Level of Evidence and Clinical Maturity

Diagnostic, educational, and patient monitoring AI applications demonstrate strong clinical maturity. While educational chatbot evaluations rely on Level 1 feasibility studies [37] and patient and educator surveys rest on Level 2 observational data [35,36,41], the field is supported by robust Level 3 randomized controlled trials (RCTs) and systematic reviews [38,39,40]. Specifically, RCTs and systematic reviews confirm that AI significantly enhances clinical trial patient recruitment [38], overall patient satisfaction [40], and post-treatment orthodontic compliance [39], driving diagnostic and educational AI toward routine clinical implementation (Level 4).

#### 3.1.4. Dentoalveolar and Periodontal Surgery

Periodontics remains a field of dentistry underappreciated by patients (and doctors). Yet, it is largely a general indicator of oral health. It relies on extensive quantitative data, i.e., probing depths, presence or absence of bleeding on probing (BOP), furcation involvement, or the presence of horizontal and vertical bone defects. This complexity makes it an ideal candidate for computational analysis [42].

Naturally, as mentioned above, radiological analysis for periodontal health is the most obvious application of AI in this area. Automatic marking of bone levels on panoramic X-rays [43] would draw greater attention from patients (and doctors) to early-stage periodontal disease. By identifying the cementoenamel junction (CEJ) of each tooth, the algorithm can instantly calculate the percentage of bone loss relative to root length and inform the clinician about disease progression. Clear, quantitative data serve as a transparent indicator for the patient of whether treatment is needed.

Likewise, on 3D images, automatic segmentation of bone defects [44] into horizontal and vertical could contribute to a better understanding of therapeutic options. Vertical defects, which can be regenerated, might be consulted faster by a periodontist, while horizontal crater-type defects, where regeneration is pointless, would fundamentally not be considered for regenerative periodontal treatment.

Additionally, furcation involvement, the presence of periapical lesions [45], or crestal resorption [46] could be automatically detected by artificial intelligence every time a CBCT/OPG (orthopantomogram) /periapical X-ray is taken. According to Calazans M., the accuracy of AI in assessing periapical lesions on CBCT reaches 70% [47]. This seems particularly significant in the context of assessing odontogenic foci of infection. Many systemic diseases are rooted in oral diseases. Automatic identification of regions “suspicious” for anomalies would act as an indicator for general practitioners, specialists in other fields (not dealing with the mouth or teeth), and junior clinicians, for whom AI support significantly improved overall diagnostic accuracy from 91.6% unassisted to 93.3% with AI support—mainly by reducing false-positive diagnoses [48]. Identifying dental abnormalities is often the answer when searching for the source of persistent fever, cardiological diseases, immunological conditions, or chronic inflammation manifesting in laboratory tests.

The foundation of periodontal treatment is a proper diagnosis [49]. For this purpose, it is necessary to assess the stage, i.e., the severity of the periodontal disease (I-IV), and the grade, i.e., the risk of progression (A–C). Staging requires evaluating clinical attachment loss via probing depth, or, in the absence of such information, evaluating radiographic bone loss. Automatically determining the stage based on radiological images would definitely direct the treatment process immediately, i.e., stages III and IV require implementing surgical treatment. However, to determine the grade, it is best to evaluate radiographs taken several years apart or—if prior radiological records are unavailable—the percentage of bone loss relative to the patient’s age. Archiving multiple images and comparing them quantitatively is a perfect task for AI. Moreover, the automatic integration of radiological data with medical records in the form of modifying factors, such as HbA1c levels, smoking, and patient age, would ensure standardized patient assessment and reduce the doctor’s administrative burden.

While analyzing radiological images is essentially analyzing the history of a tooth or group of teeth, analyzing intraoral photographs assesses the situation “here and now,” most often within the gums themselves. Machine learning algorithms trained on intraoral photos using, for example, fluorescence, can detect subtle soft tissue changes preceding measurable clinical attachment loss. Classification studies have shown accuracy ranging from 0.46 to 1.00, detection studies from 0.56 to 0.78, and segmentation studies achieved results from 0.43 to 0.70 [50]. As Mao notes, standardizing evaluation and reporting metrics is crucial.

Artificial intelligence can also support the home, daily hygiene phase for periodontal patients. Yuan Li [51] indicated an almost 8% better plaque control, and thus periodontitis control, in patients using toothbrushes with multimodal sensors capable of guiding patients in real-time regarding oral hygiene and transmitting valuable data to doctors, thereby enabling effective remote monitoring and guidance. Optimizing patient plaque control pre-operatively using these AI tools can significantly reduce the bacterial load, thereby lowering the risk of post-surgical site infections, impaired wound healing, or peri-implantitis.

Periodontics acts as a kind of bridge between dentistry and general medicine. It is proven that patients with periodontal disease are at higher risk of developing diabetes, cardiovascular diseases, or having low birth weight infants. Data mining large databases can establish new cause-and-effect relationships between periodontal health and other systemic diseases.

##### Level of Evidence and Clinical Maturity

Periodontal and dentoalveolar AI applications demonstrate strong clinical maturity. While CBCT lesion classification and feature segmentations rely on Level 1 proof-of-concept studies [45,47] and Level 2 retrospective data [43,49], this field is supported by robust Level 3 randomized controlled trials and systematic reviews [42,44,50]. Specifically, RCTs confirm that AI decision support significantly improves clinician diagnostic accuracy [48] and that AI-guided hygiene tools deliver superior periodontitis control [46,51], driving periodontal AI toward clinical implementation (Level 4).

In summary, the Table 1. below depicts the main AI applications that could be implemented step by step in oral surgery. The main type of artificial intelligence used and the level of evidence are indicated.

### 3.2. Maxillofacial Surgery—Oncology, Maxillofacial Trauma and Orthognathic Surgery

Maxillofacial surgery is a broad field dominated by oncology, trauma surgery, and orthognathic surgery combined with plastic surgery. Implementing the power of artificial intelligence in this field is not meant to exclude or replace the scalpel, but to streamline the decision-making process. For instance, the screening evaluation of panoramic radiographs after maxillofacial trauma to rule out mandibular fractures [52] can be helpful for general practitioners.

#### 3.2.1. Oncology

Squamous cell carcinoma (SCC) remains the most prevalent and challenging malignancy in maxillofacial oncology. Survival rates largely depend on early detection, well-planned surgical intervention, and the achievement of clear resection margins. Currently, artificial intelligence is being integrated into three key stages of the oncological treatment process.

##### Preoperative Stage

As mentioned above, the analysis of radiological images and their screening for oral tumors is one of the primary applications of AI. In oncological diagnostics, evaluating the biopsy specimen and subsequently establishing the correct diagnosis is of equal importance. This is usually the bottleneck of oncological treatment—the average waiting time for histopathology results of head and neck specimens, e.g., at the NHS Manchester University from July to August 2025, is 26.56 days. As reported by S.Y. Jang [53], using AI resources during the evaluation of histopathological slides helps reduce the assessment time by almost 6.5 min for a junior pathologist. Furthermore, it was also shown that deep learning models can improve image classification accuracy, thereby reducing human error and workload for both junior and senior clinicians. Meanwhile, Mohanad A. Deif [54] achieved a classification accuracy of 96.3% for biopsies containing squamous cell carcinoma cells—he draws attention to another important aspect of using deep learning models: cost reduction with less susceptibility to human error. Kumar Das, in turn, achieved an efficacy of 96.88% in detecting keratin pearls during the automated identification of oral squamous cell carcinoma histopathology slides [55].

Moreover, artificial intelligence can accurately predict the occurrence of oral cancer based on the collected medical history compared to conventional methods by analyzing predisposing factors such as age, gender, tobacco smoking, and biomarkers [56]. Sanjeev B. Khanagar reports that the precision and accuracy of AI in diagnosis, as well as in predicting occurrence, are higher than those of current clinical strategies and conventional statistics.

Another area of interest is the use of radiomic features from magnetic resonance imaging (MRI) scans—extracted via machine learning methods—to detect occult cervical lymph node metastases in early-stage oral cavity cancer (OCC) [57]. Assessing the tumor staging is crucial for treatment planning, as it helps avoid undertreatment in the presence of metastases or overtreatment when there is no lymph node involvement.

##### Intraoperative Stage

The main dilemma in resection is striking a balance between radicality and preserving functionality. AI-assisted segmentation—as Manea Alahmari notes [58]—is characterized by a high accuracy in segmenting dental and maxillofacial structures, comparable to professional manual segmentation; sensitivity in the analyzed studies exceeded 90%. AI also segments tumors from healthy tissue on MRI and computed tomography (CT) scans. Software proposes optimal osteotomy angles to reconstruct the contour of the mandible and maxilla and restore occlusion, reducing virtual surgical planning time from hours to minutes.

Intraoperatively, new technologies allow for real-time visualization of tumor margins during surgery, thereby reducing the rate of positive margins. Although this solution is not yet in widespread use [59], robotic techniques are more frequently employed in maxillofacial surgery due to the extensive nature of the procedures and the larger surgical field compared to oral surgery procedures. They also allow for a small, less invasive approach.

##### Postoperative Stage

Equally as important as early and correct diagnosis, rapid diagnostics, precise surgical execution, and proper reconstruction of the postoperative defect are follow-up visits scheduled according to the oncological treatment protocol and the performance of follow-up CT scans at the right frequency. AI seems to be an ideal candidate for typical administrative tasks. Guiding the patient through follow-up visits, maintaining appropriate intervals between them, and scheduling appointments according to recommendations can be easily automated, thereby keeping postoperative patient monitoring free from typical clerical errors. Early detection of potential recurrence greatly increases the chances for reoperation, easier reconstruction, and keeping the patient alive and healthy longer [60,61].

##### Level of Evidence and Clinical Maturity

Maxillofacial oncology AI applications demonstrate evolving translational readiness across diagnostic and surgical stages. While intraoperative tumor margin visualization, AR cutting guides, and virtual surgical planning rely on Level 1 preclinical models [59], and histopathological classification algorithms rest on Level 2 retrospective datasets [53,54,55], the domain is anchored by Level 3 systematic reviews [56,58,61]. Specifically, these syntheses confirm high accuracy in oral malignancy risk prediction and anatomical segmentation [57,58], as well as improved postoperative follow-up adherence [60,61].

#### 3.2.2. Maxillofacial Trauma

Maxillofacial trauma represents a substantial portion of emergency department admissions. Because facial injuries often present alongside polytrauma, the initial radiological assessment is frequently performed by emergency physicians or general radiologists rather than maxillofacial specialists. Due to the complex overlapping anatomy of the facial skeleton, fractures—particularly subtle, non-displaced ones—are easily overlooked. Consequently, artificial intelligence has emerged as a highly effective triage and diagnostic tool in this domain, significantly reducing the risk of missed diagnoses.

##### Diagnostic and Triage Stage

The primary application of AI in maxillofacial trauma lies in the automated detection of fractures using CNNs. Mandibular fractures are the most common injuries in the facial skeleton and are typically initially screened using panoramic radiographs. While simple and accessible, panoramic X-rays are subject to distortion and superimposition (e.g., the cervical spine overlapping the symphysis). Recent deep learning models have demonstrated remarkable efficacy in overcoming these limitations. CNNs trained on thousands of panoramic images can automatically highlight radiolucent fracture lines and cortical discontinuities. Studies indicate that AI models can achieve accuracy, sensitivity, and specificity exceeding over 90% in detecting mandibular fractures, performing on par with, and sometimes surpassing, junior clinicians and general practitioners [62].

For more complex midface and orbital injuries, Computed Tomography (CT) and Cone-Beam CT (CBCT) are the gold standard. Here, 3D CNNs are utilized to navigate the intricate anatomy of the zygomaticomaxillary complex (ZMC), naso-orbito-ethmoid (NOE) region, and orbital walls. AI algorithms can rapidly analyze multi-planar CT slices to identify maxillofacial fractures [63]. By acting as a “second read” for the on-call physician, AI significantly reduces the bottleneck in emergency settings, ensuring that patients are promptly referred to the maxillofacial surgery department.

##### Preoperative Planning and Reconstructive Stage

Beyond simple detection, in severe comminuted pan-facial fractures, AI-assisted virtual surgical planning (VSP) algorithms can mirror healthy contralateral anatomy to virtually reconstruct shattered bone segments. This allows for the precise, automated pre-bending of patient-specific titanium implants or the milling of orbital mesh prior to the patient even entering the operating room [64]. By streamlining both the diagnostic triage and the reconstructive planning, AI effectively reduces operative time and improves esthetic and functional outcomes for trauma patients [65].

##### Level of Evidence and Clinical Maturity

Maxillofacial trauma AI applications demonstrate strong translational readiness across emergency triage and reconstructive stages. While deep learning models for panoramic and CT fracture detection rely on Level 2 retrospective validation datasets [62,63], reconstructive planning and custom implant fabrication are supported by Level 3 comparative case–control studies and systematic reviews [64,65].

#### 3.2.3. Orthognathic Surgery

Orthognathic surgery is a combination of maxillofacial surgery and orthodontics. The goal is singular: restoring facial function and esthetics by translating numbers obtained via cephalometry into the functional and esthetic results desired by the patient.

Cephalometric analysis—as reported by Julie Hendrickx [66]—takes an average of less than a minute when performed by AI, and landmark detection on 2D images is done with an error below the clinically acceptable threshold of 2 mm. Therefore, tedious data collection can be successfully performed by AI, while drawing subsequent conclusions (and taking responsibility for them) can remain the doctor’s domain. O.M. Tanaka [67] went further, comparing treatment plans generated by AI from different providers with a plan created by an orthodontist, which was then evaluated by dozens of orthodontists. Doctors showed a significantly higher level of agreement with the treatment plans proposed by the orthodontist compared to plans generated by AI platforms. This is likely a temporary technical limitation of AI; nonetheless, the stage where models are capable of collecting and segregating data seems to have already arrived.

Furthermore, the application of convolutional neural networks (CNNs) during orthognathic surgery may reduce segmentation time from 7 h to 5 min and allow the analysis of condylar morphology, comparing the outcomes of the surgery-first approach (SFA) with the traditional surgery-late approach (SLA). Combining SFA with AI does not increase the side effects of condylar remodeling compared to SLA; on the contrary, it supports safety during surgical procedures and reduces orthodontic treatment time [68].

Undoubtedly, the root cause of a patient starting orthodontic treatment is the desire to improve their appearance, achieve a better esthetic smile, and improve overall facial perception—in short, achieving dreamed dentofacial esthetics. Obwegeser D. [69] used AI in her research to determine the impact of dental esthetics on facial attractiveness compared to other facial modifications; she noted that orthodontic intervention enhances perceived facial attractiveness to a degree comparable to the application of cosmetic makeup. For a patient deciding to begin orthodontic treatment, the visualization of the face, lip posture, and nose, rather than just the teeth—especially regarding comprehensive orthognathic surgical treatment—seems to be a tremendous added value.

Orthognathic surgery also includes assessing airway volume [70] or temporomandibular joint disorders [71]. Artificial intelligence can help segment specified structures and assist the doctor in clinical decision-making.

##### Level of Evidence and Clinical Maturity

While automated treatment planning, condylar morphology analysis, and facial esthetic scoring rely on Level 1–2 pilot studies and retrospective data [68,69], the domain is firmly anchored by Level 3 systematic and umbrella reviews [67,71]. Specifically, high-level evidence validates sub-2 mm accuracy in AI-driven cephalometric landmark tracing [66] as well as precise CBCT airway and TMJ volumetric analysis [70,71], advancing orthognathic AI close to routine clinical implementation.

The Table 2. below depicts the main AI applications in the field of maxillofacial surgery that could be implemented during a patient’s treatment. The main type of artificial intelligence used and the level of evidence are indicated.

## 4. Discussion

Artificial intelligence is rapidly integrating into every area of life, including diverse healthcare sectors. Its implementation in medicine, specifically here in dental and maxillofacial surgery, is no longer a question but a fact. There are constantly new estimations regarding how many professions will simply disappear from the job market. This also applies to the medical sector. It seems that the hardest jobs to replace will be those requiring manual work and direct human contact—i.e., with the client/patient. Nevertheless, artificial intelligence represents a paradigm shift in clinical practice, comparable (or even greater?) to the digitization of medical records.

The difference is that AI is like the Internet of the 2000s but with the ability to draw conclusions, connect facts, and form hypotheses—and that sounds like a perfect tool and guidepost for both doctor and patient and has a strong potential as a clinical adjunct.

Certainly, dental and maxillofacial surgeons can streamline their work, which involves elements of deduction, planning, and manual precision, thanks to it. Tomlinson K. [72] lists the maxillofacial surgeon profession as one of the 40 jobs with the lowest AI applicability score.

Undoubtedly, the work optimization achievable through artificial intelligence is a massive advantage given the aging population, difficulties for young doctors to specialize, staff shortages in smaller towns, and the transportation exclusion of people living far from large urban centers.

However, while the theoretical and preclinical potential of AI is vast, the transition from proof-of-concept to routine clinical reality requires overcoming significant methodological, ethical, and regulatory hurdles.

### 4.1. Critical Evaluation of Current Evidence and Generalizability

A critical appraisal of the current literature reveals that much of the enthusiasm surrounding AI in maxillofacial surgery is mostly based on preliminary or retrospective data. Many of the cited studies—particularly those evaluating convolutional neural networks for fracture detection, anatomical landmarking, and histopathological screening—rely on relatively small, single-center datasets.

This reliance on localized data introduces a significant risk of bias, particularly selection and algorithmic bias. AI models trained on homogeneous populations or standardized imaging equipment from a single institution often suffer from “overfitting,” meaning they perform exceptionally well on their training data but fail when exposed to real-world clinical variations. Consequently, the generalizability of these models remains a major limitation. Until these algorithms undergo rigorous external validation in prospective, multi-center trials involving diverse patient demographics and varying data acquisition protocols, the high accuracy rates (often reported at >90%) must be interpreted with clinical caution.

### 4.2. Regulatory Barriers to Clinical Implementation

Beyond methodological limitations, significant regulatory barriers impede the widespread clinical integration of AI. For an AI model to be utilized in daily surgical workflows, it must typically be classified and approved as “Software as a Medical Device” (SaMD) by regulatory bodies such as the FDA [73] (in the USA) or under the Medical Device Regulation (MDR) in Europe. The stringent requirements for SaMD certification demand rigorous, continuous proof of clinical safety, efficacy, and post-market surveillance.

### 4.3. Ethical, Legal, and Security Challenges

Firstly, the issue of responsibility for potential errors during the diagnostic and treatment process remains a question. The “black box” phenomenon is inherently linked to large language models and imaging networks. A surgeon can plan an implant position, determine an osteotomy scope, or qualify teeth for extraction during oral cavity sanitation by considering the patient’s objective and subjective examinations. The answer to the “black box phenomenon” is the creation of explainable AI (XAI), where one can trace the reasoning process of the chosen language model. The question remains, however: in the event of an error during the diagnostic or planning stage, who bears responsibility toward the patient?

Secondly, AI performance is strictly tied to access to training data. There is a risk that a model trained on limited data may subsequently be responsible for systemic biases. Diversifying training data, however, is time-consuming, expensive, and technically complex.

Related to the above is the third point: data privacy and cybersecurity. The development of AI in medicine requires vast amounts of private patient data, which poses a significant security risk if centralized. To mitigate these concerns, federated learning is rapidly emerging as a primary technological solution. In a federated learning framework, the AI model is trained locally on individual hospital or clinic servers. The algorithm learns and updates its parameters, but the raw patient data never leaves the institution. This decentralized approach allows for the collaborative creation of robust, multi-center AI models while strictly preserving patient confidentiality and minimizing vulnerability to cyberattacks. Furthermore, utilizing these AI models in clinical settings will require recognizing them as formal medical tools, subject to stringent security measures much like enterprise cloud accounts or corporate emails, thereby creating a crucial space for future legislative and governmental regulation.

### 4.4. Limitations

The following limitations apply to this review. First, although the literature search encompassed major databases (PubMed/MEDLINE, Scopus, Web of Science), the inherent design of a narrative review means the search strategy was not strictly exhaustive or systematically documented, which may introduce selection bias and limit reproducibility. Due to the type of the article (narrative review), the search strategy is not strictly documented. The chosen format of the article allowed us to describe what is known on a topic while conducting a subjective examination, but it is burdened by the lack of reproducibility. Rather than a systematic appraisal of all AI algorithms, the literature search deliberately focused on studies demonstrating the practical integration and future potential of AI in daily surgical workflows. Furthermore, this narrative review is also the beginning of the article cycle focusing on the application of artificial intelligence in oral surgery.

Second, a key limitation identified in the current clinical AI literature—and relevant to the translation of these tools into oral and maxillofacial surgery—is the variability in algorithm reporting and validation quality. To ensure clinical safety, generalizability, and rigorous appraisal, future research and clinical implementations must adhere to established standardized reporting and validation frameworks. These include STARD-AI for diagnostic accuracy studies, TRIPOD-AI for developing and validating prediction models, and CONSORT-AI for clinical trials evaluating AI-based interventions [74]. There are also other reporting guidelines like: CLAIM, DECIDE-AI or CHEERS-AI. Widespread adoption of these frameworks is essential to address algorithmic bias, ensure data transparency, and establish reliable clinical validation before widespread clinical integration.

Finally, artificial intelligence is not meant to replace the maxillofacial surgeon; it is meant to support them, facilitate their work, and save their time. An algorithm cannot build a trust-based bond with a patient, share empathy with a sick person, or incorporate palpation into a comprehensive patient assessment. Instead, it should serve as an assistant, a second opinion—filtering data, making summaries, and proposing solutions. The ultimate decision, however, along with the responsibility for the treatment process, should be made by the attending physician.

## 5. Conclusions and Future Directions

The use of artificial intelligence in dental and maxillofacial surgery is no longer a futuristic concept; it is a clinical reality. Radiological diagnostics, guided implantology, qualification for dental clearance, tumor resections, or osteotomy boundaries in orthognathic surgery—in all these areas, AI is already providing support to the surgeon. By increasing diagnostic accuracy and optimizing surgical efficiency, more predictable outcomes for patients can be achieved. Although AI applications such as the use of augmented reality or robotic surgery—during the “surgical” part of the procedure—are still rather a technical exception, rather than a common solution, this might change in the future.

However, to fully harness the potential of large language models and imaging networks, it is necessary to create multi-center, large databases with full patient data anonymization and to develop XAI infrastructure. At the institutional level, integrating this XAI into multidisciplinary surgical boards will require algorithms to output probabilistic data—such as providing a confidence percentage for inferior alveolar nerve proximity—rather than binary ‘yes/no’ decisions. Such transparent ‘confidence scores’ will ensure that surgeons can appropriately weigh algorithmic recommendations alongside their own clinical judgment. Furthermore, university and specialization curricula should include the development of digital competencies regarding cooperation with AI and its critical evaluation.

It is clear there is no justification for fear of this technological revolution. On the contrary, it should be integrated into the professional environment, utilized, and critically assessed based on experience. Through the synergistic action of human experience and artificial intelligence, the boundaries of what is possible in maxillofacial surgery can be pushed, ushering in a new era of precision, safety, and predictability in patient care.

## Figures and Tables

**Table 1:** AI application in oral surgery.

Field of Oral Surgery	Specific Clinical Application	Type of AI Utilized	Level of Evidence (Mostly)	Primary Clinical Benefit	Source
Extraction	Risk assessment and difficulty calculation for impacted third molars.	CNN & Multimodal AI	Retrospective Validation (Level 2)	Provides objective, quantitative risk assessment for inferior alveolar nerve injury. Automates “difficulty index” calculation to optimize surgical booking time and clearly visualizes surgical risks for patient consent.	[14,15,16,17,18,19]
Implantology	Anatomical landmark detection, bone density analysis, and brand recognition.	CNN & Multimodal AI	Retrospective Validation & Systematic Reviews (Level 2–3)	Reduces surgical planning time from 30 to 10 min. Prevents iatrogenic injury by auto-segmenting the maxillary sinus and mandibular canal. Predicts patient-specific risk for peri-implantitis or osseointegration failure.	[26,27,28,29,30,32,33]
Radiological diagnosis	Triage screening for periapical lesions, and cysts on 2D/3D images.	CNN	Retrospective Validation (Level 2)	Acts as a “radiological bridge” between specialists. Standardizes initial screenings, reduces false-positive diagnoses by junior clinicians (up to 93.3% accuracy), and highlights lesions visually to increase patient engagement.	[35,36,37,38,48]
Postoperative care	Remote patient monitoring and postoperative communication.	LLM	Prospective Validation & Clinical Trials (Level 3)	Provides 24/7 triage for patient concerns regarding healing, pain, or hygiene. Significantly reduces the surgeon’s administrative burden while minimizing patient anxiety and improving compliance.	[21,25,39,40,41]
Periodontology	Detection of alveolar bone loss and real-time plaque control.	CNN & Multimodal AI	Retrospective & Prospective RCT Validation (Level 2–3)	Automatically calculates the percentage of bone loss relative to root length. Speeds up staging/grading by quantitatively comparing new radiographs with archived ones. Guides real-time daily home hygiene.	[42,43,44,45,46,47,48,49,50,51]

**Table 2:** AI application in maxillofacial surgery.

Field of Maxillofacial Surgery	Specific Clinical Application	Type of AI Utilized	Level of Evidence (Mostly)	Primary Clinical Benefit	References
Oncological diagnosis	Automated evaluation of histopathology slides for squamous cell carcinoma (SCC).	CNN & Multimodal AI	Retrospective validation (Level 2)	Eliminates the diagnostic bottleneck by reducing assessment time for junior pathologists by up to 6.5 min per slide. Achieves over 96% accuracy in identifying keratin pearls and SCC cells, accelerating the start of treatment.	[53,54,55,56,57]
Oncologic surgery	Tumor margin visualization and optimal osteotomy planning.	CNN	Proof-of-Concept & Preclinical (Level 1)	Achieves >90% sensitivity in separating tumors from healthy tissue on MRI/CT. Reduces virtual surgical planning from hours to minutes while calculating optimal osteotomy angles to preserve functional anatomy.	[58,59]
Oncological follow-up	Tracking and scheduling post-resection follow-up protocols.	LLM	Systematic review & Clinical Implementation (Level 3–4)	Eliminates clerical scheduling errors and ensures strict adherence to required oncological follow-up intervals, significantly increasing the probability of early recurrence detection.	[60,61]
Maxillofacial trauma	Automated fracture detection on radiographs/CT, and Virtual Surgical Planning (VSP) for custom implants.	CNN	Retrospective & Prospective Validation (Level 2–3)	Acts as a reliable “second read” (achieving >90% accuracy for subtle fractures). Reduces operative time by enabling automated pre-bending of patient-specific titanium implants and orbital meshes.	[62,63,64,65]
Orthognathic surgery	Cephalometric analysis and facial soft-tissue prediction.	CNN	Proof-of-Concept & Retrospective Validation & Prospective Validation (Level 1–2–3)	Performs tedious 2D cephalometric landmarking in under one minute with an error margin below 2 mm. Generates predictive 3D models of postoperative facial esthetics to align surgical outcomes with patient expectations.	[66,67,68,69,70,71]

## Data Availability

No new data were created or analyzed in this study. Data sharing is not applicable to this article.

## Abbreviations

The following abbreviations are used in this manuscript:

AI	Artficial intelligence
AR	Augmented reality
BOP	Bleeding on probing
CBCT	Cone beam computed tomography
CEJ	Cementoenamel junction
CNN	Convolutional neural network
CT	Computed tomography
FDA	Food and drug administration
LLM	Large language model
MDR	Medical device regulation
MRI	Magnetic resonance imaging
NOE	Naso-orbito-ethmoid
OCC	Oral cavity cancer
OMFS	Oral and maxillofacial surgery
RCT	Randomized controlled trials
SAMD	Software as a medical device
STL	Standard triangulation language
VSP	Virtual surgical planning
XAI	Explainable artificial intelligence
ZMC	Zygomaticomaxillary complex
